# 3.0 T MRI is more recommended to detect acetabular labral tears than MR Arthrography: an updated meta-analysis of diagnostic accuracy

**DOI:** 10.1186/s13018-022-02981-1

**Published:** 2022-03-01

**Authors:** Peng Zhang, Chunbao Li, Wenliang Wang, Baiqing Zhang, Weicheng Miao, Yujie Liu

**Affiliations:** 1 Department of Sports Medicine, Characteristic Medical Center of Chinese People’s Armed Police Forces, No. 220, Chenglin Road, Tianjin, 300162 People’s Republic of China; 2 Chinese PLA Medical School, No. 28, Fuxing Road, Beijing, 100853 People’s Republic of China; 3 Department of Orthopedics Surgery, Chinese PLA General Hospital, No. 28, Fuxing Road, Beijing, 100853 People’s Republic of China

**Keywords:** Hip injuries, Magnetic resonance imaging, Diagnosis, Arthrography, Meta-analysis

## Abstract

**Background:**

This meta-analysis aimed to evaluate the current evidence on the diagnostic performance of MRI/MRA for detecting acetabular labral tears (ALT).

**Methods:**

We systematically searched the PubMed, Embase, and Cochrane library until February 5, 2021, to identify original research studies reporting the diagnostic performance of MRI/MRA for the detection of ALT. Study methodological quality was assessed using the Quality Assessment of Diagnostic Accuracy Studies 2 (QUADAS-2) tool. The summary sensitivity (Se) and specificity (Sp) of the studies were estimated using a bivariate model. We calculated the post-test probability to assess the clinical utility of MRI/MRA. Univariate meta-regression and subgroup analyses were performed to assess between-study heterogeneity.

**Results:**

We included 22 studies (*n* = 1670 patients). The meta-analytic summary Se and Sp for MRI were 0.8 (95% CI 0.51–0.94) and 0.77 (95% CI 0.68–0.84), respectively, while for MRA they were 0.89 (95% CI 0.82–0.93) and 0.69 (95% CI 0.56–0.80). MRA showed a higher area under the summary receiver operating curve (SROC) (0.87 vs. 0.80) than MRI. MRI could increase the post-test probability to 0.78 and could decrease the post-test probability to 0.21, MRA could increase the post-test probability to 0.74 and could decrease the post-test probability to 0.14. Meta-regression analysis showed two significant factors affecting study heterogeneity: MR field strength and reference standard. After dividing the studies into two subgroups based on the MR field strength, we found that the Se values of 3.0 T MRI were very close to MRA (0.87 vs. 0.89), the Sp values of 3.0 T MRI were superior to MRA (0.77 vs. 0.69).

**Conclusions:**

Given that 3.0 T MRI could provide a non-invasive, fast and convenient method to recognize suspicious ALT cases, 3.0 T MRI is more recommended than MRA.

## Introduction

Acetabular labrum is a fibrocartilaginous structure that lines the majority of the acetabular socket. The hip labrum has many functions, including shock absorption, joint lubrication, pressure distribution, and aiding in stability. Acetabular labral tears (ALT) were observed in 62% of individuals with hip or groin pain and 54% of asymptomatic individuals [1]. Five categories of ALT have been described based on etiology: traumatic, congenital, degenerative, capsular laxity, and idiopathic [2]. FAI is one of the primary predisposing factors to ALT.

Diagnosis of ALT is based on a dedicated examination of patient history, pertinent objective findings, special clinical tests, and supportive imaging findings. It is generally believed that early surgical intervention of acetabular labral tears may delay the development of osteoarthritis. Thus, the diagnostic information of ALT would have a significant effect on an orthopedic surgeon’s clinical decision-making considering surgical intervention. The use of MRI as a non-invasive, fast and convenient test to diagnose ALT has gained in popularity. However, there are some areas (e.g., the shoulder, the wrist, the hip) in which evaluation of the joint space may be suboptimal [3]. To address these issues, contrast materials may be injected into the hip joint space to perform MR arthrography (MRA), creating distention of the joint.

To be useful to clinicians, a diagnostic test must possess high sensitivity (Se) to rule in a condition and high specificity (Sp) to rule out a condition. However, there has been shown that both conventional MRI and MRA at field strengths of 1.5–3.0 T achieve different Se and Sp in detecting hip labral tears when compared to either arthroscopic or open surgical findings [4–25]. At the same time, the diagnostic accuracy of 1.5 T and 3.0 T MRI for detecting labral tears is also different, and there is no conclusive conclusion that which field strengths should be recommended. Thus, in clinical practice, whether high field MRI has the potential to substitute MRA deserves extensive discussion.

The purpose of this study was to determine (1) the diagnostic accuracy of MRI and MRA for the detection of ALT, (2) whether 1.5 T or 3.0 T is all acceptable, by conducting a meta-analysis of the literature regarding the diagnostic performance of MRI/MRA.

## Methods

This meta-analysis strictly followed the recommendations of Preferred Reporting Items for Systematic Review and Meta-Analysis (PRISMA) guidelines [26].

### Data sources and searches

A comprehensive literature search of PubMed, Embase, and Cochrane library was performed by two researchers independently to identify all relevant studies that evaluated the diagnostic performance (Se and Sp) of MRI and MRA for the detection of acetabular labral tears. The following keywords were used: ((Acetabular labral tear OR (FAI OR Femoroacetabular impingement)) AND (Magnetic resonance imaging OR (MRI) OR (Magnetic resonance arthrography OR (MRA))) AND (diagnostic accuracy). The research lists of the relevant articles were manually searched to identify other potentially relevant articles. The search included articles published up until February 5, 2021. The studies were confined to those published in the English language.

### Eligibility criteria

After removal of duplicate articles, two researchers reviewed the identified articles to determine their eligibility according to the following criteria: (a) patients who had suspected acetabular labral tears; (b) index test, 1.5–3.0 T MRI with or without contrast agents study; (c) outcomes, diagnostic accuracy including Se and Sp for the dichotomous diagnosis of acetabular labral tears; (d) reference standard, arthroscopic or open surgical findings; and (e) clinical trials. All study subjects presented suspected primary acetabular labral tears. MRI/MRA studies with the comparison with US/CTA were included, and the data on MRI/MRA were separated from those of US/CTA. Studies were excluded according to the following criteria: (a) studies with insufficient data to allow construction of a diagnostic 2 × 2 table for imaging results; (b) review articles, letters, comments, editorials, conference abstracts, and case reports; (c) studies not in the field of interest; (d) studies involved indirect MRA for the detection of acetabular labral tears. Any disagreement was discussed and resolved by consensus.

### Data extraction and quality assessment

Using the Quality Assessment of Diagnostic Accuracy Studies 2 (QUADAS-2) criteria, two researchers independently assessed the quality of the eligible articles, including the risk of bias and the applicability of each study [27]. The disagreement was resolved by consensus. Review Manager (version 5.4, The Cochrane Collaboration) was also used to graphically display the QUADAS-2 results. The following data were extracted from each study: the first author’s name, year of publication, average age, sample size, sex distribution, study design (prospective, retrospective, or unclear), MR type, meantime MR to surgery, the reference standard, index/reference test binded design, radiologists of interpretation of the diagnostic tests and radiologists reliability (intraobserver reliability and interobserver reliability). True-positive, false-positive, false-negative, true-negative, Se and Sp results for MRI/MRA were extracted and 2 × 2 contingency tables were constructed.

### Statistical analysis

We used the bivariate random-effects model to pool the Se, Sp, and area under receiver operating characteristic curve (AUC) by using the “Midas” command [28]. Based on the parameters estimated by the bivariate model for the logit transforms of Se and Sp between the studies, we constructed a summary receiver operating characteristic curve (SROC). Also, AUC was retrieved whenever possible where AUC represented the diagnostic accuracy. The Higgins *I*^2^ statistic was used to estimate the heterogeneity among studies (*I*^2^ > 50%: substantial heterogeneity). When there was high heterogeneity, we evaluated the threshold effect through the Spearman correlation coefficient of the logarithm of Se and 1-Sp. When the *P *value was < 0.05, the threshold effect was considered significant. At the same time, we used univariate meta-regression to find the potential sources of heterogeneity, with the following variates being considered: (a) study design (retrospective vs. not retrospective); (b) MR field strength (1.5 T MR vs. 3.0 T MR); (c) index test blinded (blinded to surgery findings vs. aware of surgery findings); (d) reference standard (arthroscopic surgery vs. arthroscopic and open surgery). Then, we used the DerSimonian Laird random-effects model to conduct a subgroup analysis to pool the subgroup Se, Sp, positive likelihood ratio (LR+), negative likelihood ratio (LR-), diagnostic odds ratio (DOR) [29]. A test for publication bias (Deeks’ funnel plot) was also used to analyze the sources of heterogeneity. When the *P* value was < 0.05, the tests for publication bias were considered statistically significant.

Stata 14.0 software and Meta-DiSc 1.4 were used for data analysis.

## Results

### Search results

**Fig. 1:**
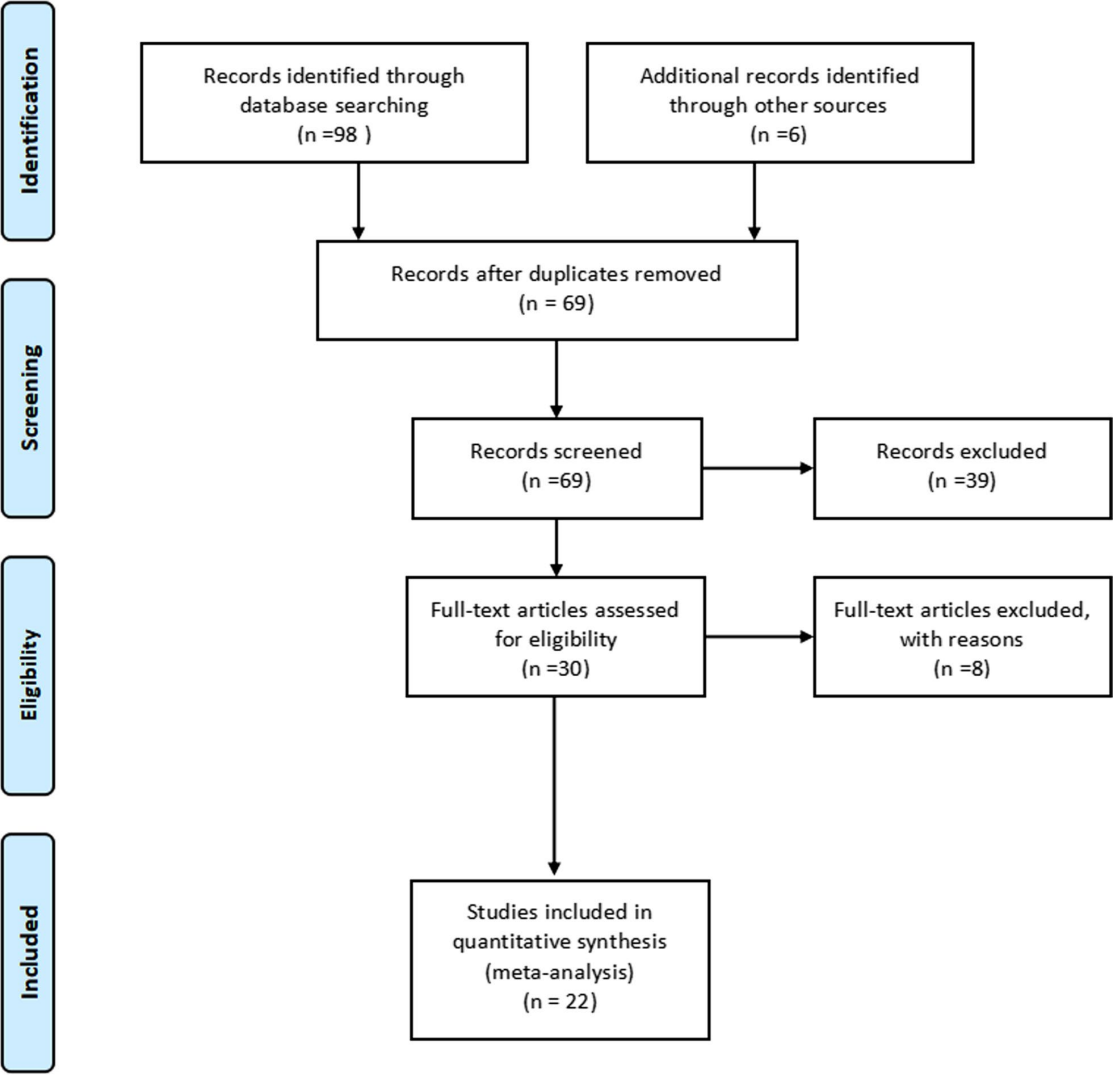
Selection process for studies included in the meta-analysis

After a systematic search in the above databases, 68 studies were initially selected, and finally, twenty-two studies were included according to the inclusion and exclusion criteria, including 1670 participants [4–25]. Of the twenty-two studies for the meta-analysis, nineteen studies reported diagnostic results for MRA [4–6, 8–21, 23, 25] and ten studies reported diagnostic results for MRI [4, 7, 9, 13, 15, 16, 20, 22–24]. One study reported the diagnostic results for MRI with a large field of view (LFV) and a small field of view (SFV) respectively [9]. The selection process and reasons other articles were excluded are described in Fig. 1.

### Study characteristics

The characteristics of included studies are summarized in Table 1. The twenty-two included studies were conducted in twelve countries. Nineteen articles were retrospective studies [4–9, 11–14, 16–24], and two were prospective studies [15, 25], one was unclear [10]. Of the nineteen MRA articles, the MR type in only two articles was 3.0 T [13, 16]. Of the ten MRI articles, the MR type in five articles was 3.0 T [13, 16, 20, 22, 24]. Arthroscopic surgery findings were used as a reference standard in eighteen articles [4–7, 9, 11–14, 16, 17, 19–25], and four articles using arthroscopic and open surgery findings together [8, 10, 15, 18].

**Table 1:** Characteristics of the included studies

Study	Country/Year	Study design	Subjects/hips	Mean age	Sex (F:M)	MR type	Mean time MR to surgery	Reference standard	Reference test blinded design	Index test blinded design	MR reviewers	Radiologists reliability
Freedman BA	USA/2006	①	24/24	37.1	13:11	MRA(1.5 T)	> 6 months	Arthroscopy	NA	Yes	1 MSK Radiologist	*k* > 0.96^a^
Leunig M	Switzerland/1997	①	23/23	40	14:9	MRA(1.5 T)	5 months	Arthroscopy	NA	NA	NA	NA
Toomayan GA	USA/2006	①	48/51	35	27:21	MRI(1.5 T) MRA(1.5 T)	NA	Arthroscopy	NA	Yes	6 MSK radiologists	NA
Chan YS	China/2005	②	30/17	41	13:17	MRA(1.5 T)	NA	Arthroscopy	NA	Yes	3 MSK Radiologists	NA
Byrd JW	USA/2004	①	40/40	NA	NA	MRI(1.5 T) MRA(1.5 T)	NA	Arthroscopy	NA	NA	1 of 2 MSK Radiologists	NA
Mintz DN	USA/2005	①	92/92	38.5	58:34	MRI(1.5 T)	NA	Arthroscopy	NA	Yes	2 MSK Radiologists	92% agreement
Studler U	Switzerland/2008	①	57/57	35	36:21	MRI(1.5 T) MRA(1.5 T)	NA	Open surgery and arthroscopy	NA	Yes	2 MSK Radiologists	*k* = 0.81^b^
Petersilge CA	USA/1996	①	10/10	38.4	5:5	MRA(1.5 T)	NA	Open surgery and arthroscopy	No	NA	1 MSK Radiologist	NA
Keeney JA	USA/2004	①	101/102	37.6	70:31	MRA(1.5 T)	NA	arthroscopy	No	NA	subspecialty radiologists	NA
Aprato A	Italy/2013	③	41/41	24.0	17:24	MRA(1.5 T,apply traction)	< 6 months	Open surgery and arthroscopy	NA	NA	Subspecialty MSK radiologists	NA
Banks DB	UK/2012	①	66/69	NA	NA	MRA(1.5 T)	< 12 weeks	Arthroscopy	No	Yes	MSK radiologist	NA
Magee T	USA/2015	①	43/43	34	15:28	MRI(3.0 T)MRA(3.0 T)	18 days	Arthroscopy	No	Yes	2 MSK radiologists	MRI:*k* = 0.88^b^ MRA:*k* = 0.85^b^
Tian CY	China/2014	①	90/90	35.1	46:44	MRI(3.0 T)MRA(3.0 T)	49.7 days	Arthroscopy	No	Yes	2 radiologists	MRI:*k* = 0.65^b^ MRA:*k* = 0.81^b^
Reurink G	Netherlands/2012	①	93/95	40.1	64:31	MRA(1.5 T)	153 day	Arthroscopy	NA	Yes	2 MSK radiologists	*k* = 0.27^b^
Crespo-Rodríguez AM	Spain/2017	①	50/50	42.5	20:30	MRA(1.5 T,apply traction) MRI(3.0 T)	8 months	Arthroscopy	No	Yes	NA	NA
Carulli C	Italy/2018	①	24/29	38.3	6:18	MRA(1.5 T)	NA	Arthroscopy	Yes	Yes	NA	NA
Annabell L	Australia/2018	①	68/71	NA	30:41	MRI(3.0 T)	< 6 months	arthroscopy	NA	Yes	2 MSK radiologists	*k* = 0.501^b^
Saied AM	Belgium/2019	①	482/490	39.5	262: 228	MRI(1.5 T) MRA(1.5 T)	NA	Arthroscopy	No	NA	1 MSK radiologist	NA
Linda DD	Canada/2017	①	38/42	29	13:25	MRI(3.0 T)	154 days	Arthroscopy	No	Yes	2 MSK radiologists	NA
Sutter R	Switzerland/2014	②	28/28	31.8	10:18	MRI(1.5 T) MRA(1.5 T)	4 months	Open surgery and arthroscopy	NA	Yes	2 MSK radiologists	MRI:*k* = 0.63^b^ MRA: *k* = 0.81^b^
Sahin M	Turkey/2014	①	14/14	35	11:3	MRA(1.5 T)	NA	Arthroscopy	NA	Yes	2 MSK radiologists	NA
Schmaranzer F	Austria/2015	①	73/75	34.5	28:45	MRA(1.5 T)(apply traction)	NA	Arthroscopy	NA	Yes	2 MSK radiologists	*k* = 0.58^b^

MR, magnetic resonance; MRA, magnetic resonance arthrogram; MRI, magnetic resonance imaging; MSK musculoskeletal; ① Retrospective study; ② Prospective study; ③ Unclear; a: intraobserver reliability; b: interobserver reliability; NA not available

### Quality assessment

**Fig. 2:**
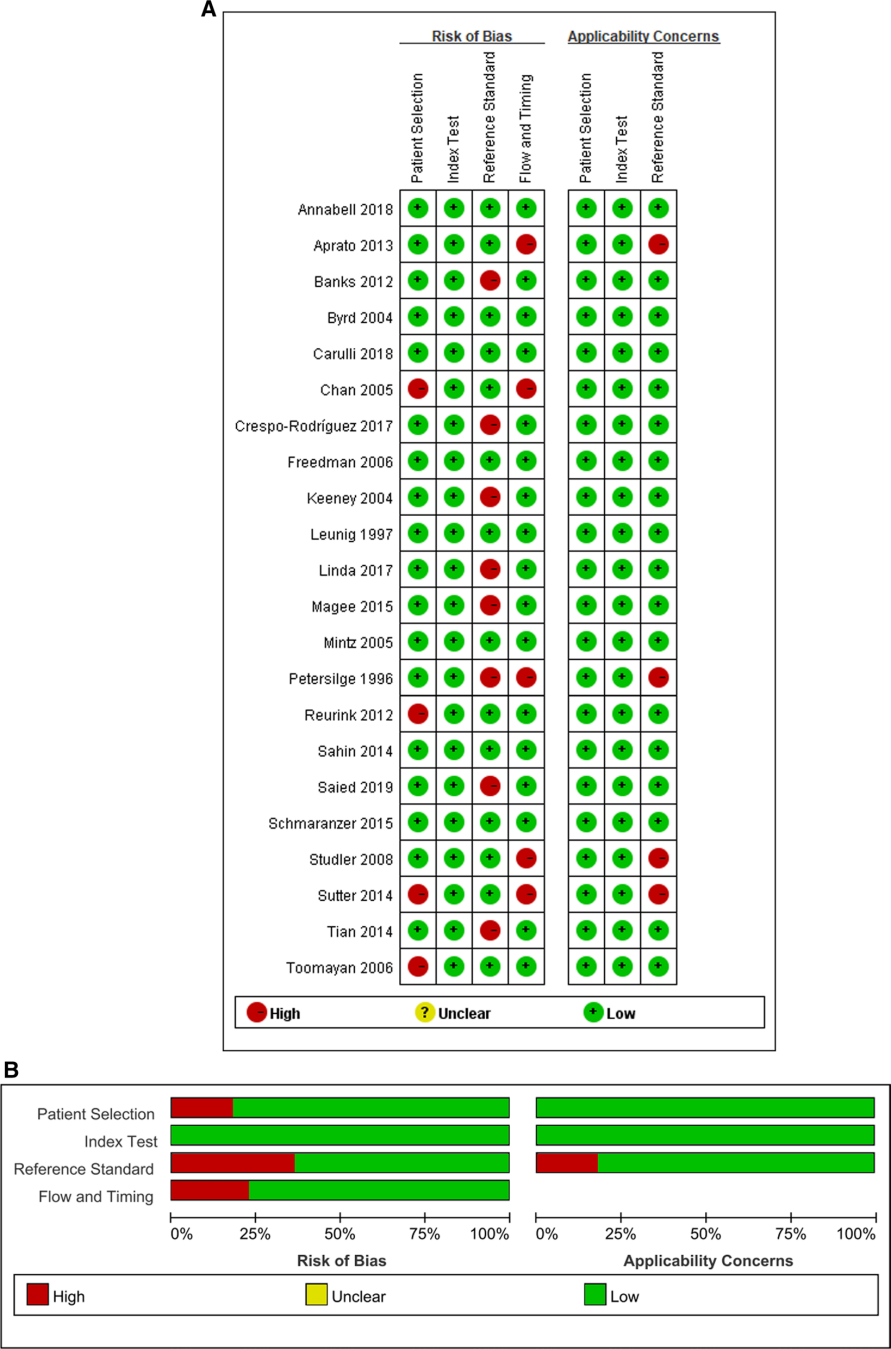
Risk of bias and applicability concerns summary (**A**). Risk of bias and applicability concerns graph (**B**)

The quality of the included articles is summarized in Fig. 2 and Table 2. Based on the QUADAS-2 criteria, most studies presented a low risk of bias and concern regarding applicability. Only one study in which the reference standard results were interpreted without knowledge of the results of the index test [21]. Besides, six studies were unclear as to whether the index test results were interpreted without knowledge of the results of surgical findings [4, 6, 10, 17, 18, 23].

**Table 2:** QUADAS-2 evaluation

Study	QUADAS Score*																
	1	2	3	Bias	Appl	4	5	Bias	Appl	6	7	Bias	Appl	8	9	10	Bias
Freedman BA	NC	1	1	Low	Low	1	1	Low	Low	1	NC	Low	Low	1	1	1	Low
Leunig M	NC	1	1	Low	Low	NC	1	Low	Low	1	NC	Low	Low	1	1	1	Low
Toomayan GA	0	1	1	High	Low	1	1	Low	Low	1	NC	Low	Low	NC	1	1	Low
Chan YS	0	1	1	High	Low	1	1	Low	Low	1	NC	Low	Low	NC	1	0	High
Byrd JW	NC	1	1	Low	Low	NC	1	Low	Low	1	NC	Low	Low	NC	1	1	Low
Mintz DN	NC	1	1	Low	Low	1	1	Low	Low	1	NC	Low	Low	NC	1	1	Low
Studler U	1	1	1	Low	Low	1	1	Low	Low	1	NC	Low	High	NC	0	1	High
Petersilge CA	NC	1	1	Low	Low	NC	1	Low	Low	1	0	High	High	NC	0	1	High
Keeney JA	1	1	1	Low	Low	NC	1	Low	Low	1	0	High	Low	NC	1	1	Low
Aprato A	NC	1	1	Low	Low	NC	1	Low	Low	1	NC	Low	High	1	0	1	High
Banks DB	NC	1	1	Low	Low	1	1	Low	Low	1	0	High	Low	1	1	1	Low
Magee T	1	1	1	Low	Low	1	1	Low	Low	1	0	High	Low	1	1	1	Low
Tian CY	NC	1	1	Low	Low	1	1	Low	Low	1	0	High	Low	1	1	1	Low
Reurink G	0	1	1	High	Low	1	1	Low	Low	1	NC	Low	Low	1	1	1	Low
Crespo-Rodríguez AM	1	1	1	Low	Low	1	1	Low	Low	1	0	High	Low	1	1	1	Low
Carulli C	1	1	1	Low	Low	1	1	Low	Low	1	1	Low	Low	NC	1	1	Low
Annabell L	1	1	1	Low	Low	1	1	Low	Low	1	NC	Low	Low	1	1	1	Low
Saied AM	NC	1	1	Low	Low	NC	1	Low	Low	1	0	High	Low	NC	1	1	Low
Linda DD	NC	1	1	Low	Low	1	1	Low	Low	1	0	High	Low	1	1	1	Low
Sutter R	0	1	1	High	Low	1	1	Low	Low	1	NC	Low	High	1	0	1	High
Sahin M	NC	1	1	Low	Low	1	1	Low	Low	1	NC	Low	Low	NC	1	1	Low
Schmaranzer F	1	1	1	Low	Low	1	1	Low	Low	1	NC	Low	Low	NC	1	1	Low
Lee GY	NC	1	1	Low	Low	1	1	Low	Low	1	1	Low	Low	NC	1	1	Low

The numbers in the top row correspond to the following questions: Domain 1: Patient selection. Numbers correspond with the following questions: (1) Was a consecutive or random sample of patients enrolled? (2) Was a case–control design avoided? (3) Did the study avoid inappropriate exclusions? Domain 2: Index test. Numbers correspond with the following questions: (4) Were the index test results interpreted without knowledge of the results of the reference standard? (5) If a threshold was used, was it pre-specified? Domain 3: Reference test. Numbers correspond with the following questions: (6) Is the reference standard likely to correctly classify the target condition? (7) Were the reference standard results interpreted without knowledge of the results of the index test? Domain 4: Flow and timing. Numbers correspond with the following questions: (8) Was there an appropriate interval between index test(s) and reference standard? (9) Did all patients receive a reference standard? (10) Were all patients included in the analysis?

^*^Number 1 indicates “yes,” and 0 indicates “no”; Bias risk: of bias; Appl.: concerns regarding applicability; NC: not clear

### Diagnostic value

#### Diagnostic accuracy of MRI for ALT

**Fig. 3:**
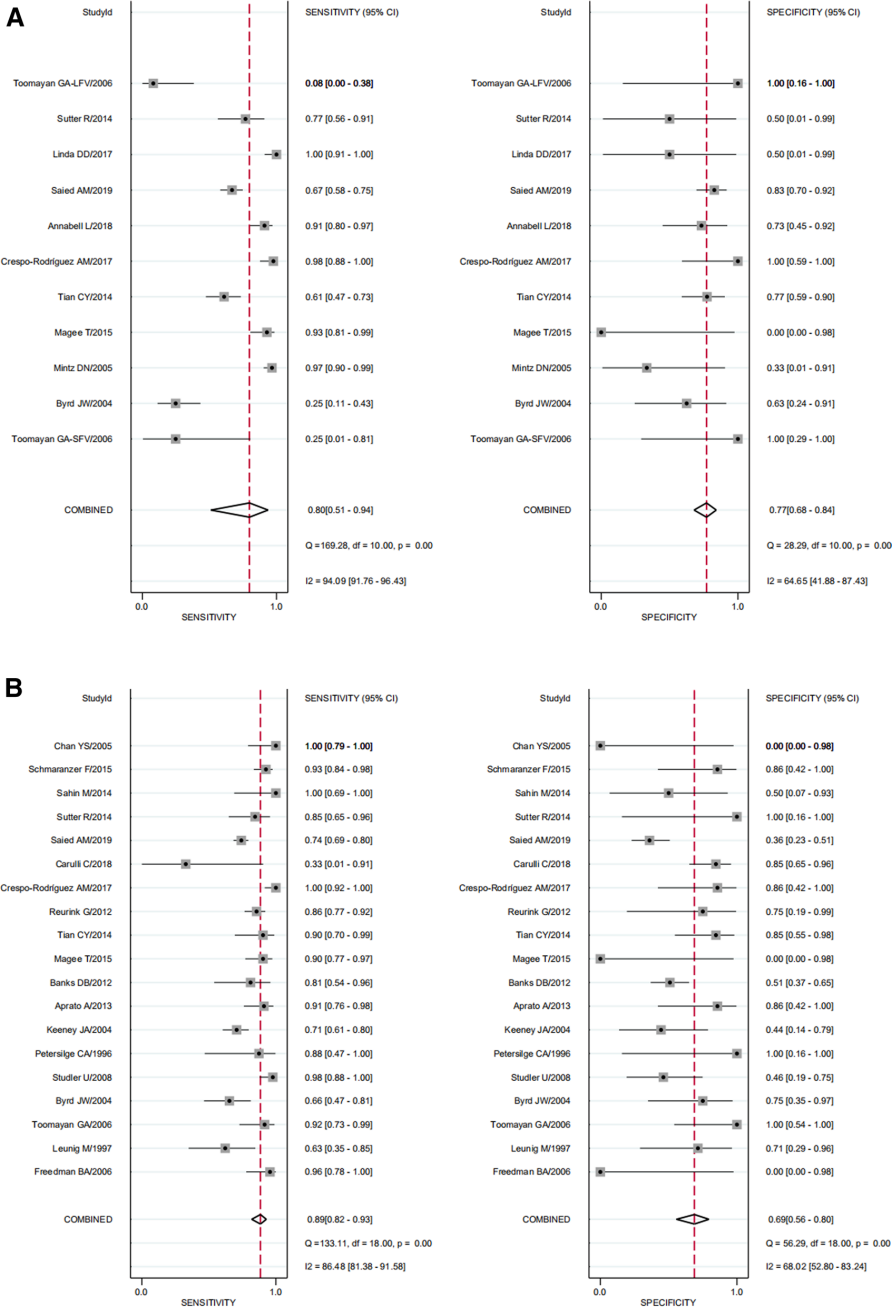
Forest plots of sensitivity and specificity. MRI (**A**), MRA (**B**)

The summary Se and Sp were 0.80 (95% CI 0.51–0.94) and 0.77 (95% I, 0.68–0.84), respectively. We found significant heterogeneity for both Se and Sp (*I*^2^ = 94.1% and *I*^*2*^ = 64.7%), which is shown in Fig. 3. The Spearman correlation coefficient was 0.460 (*P* = 0.154). The heterogeneity might not result from the threshold effects.

**Fig. 4:**
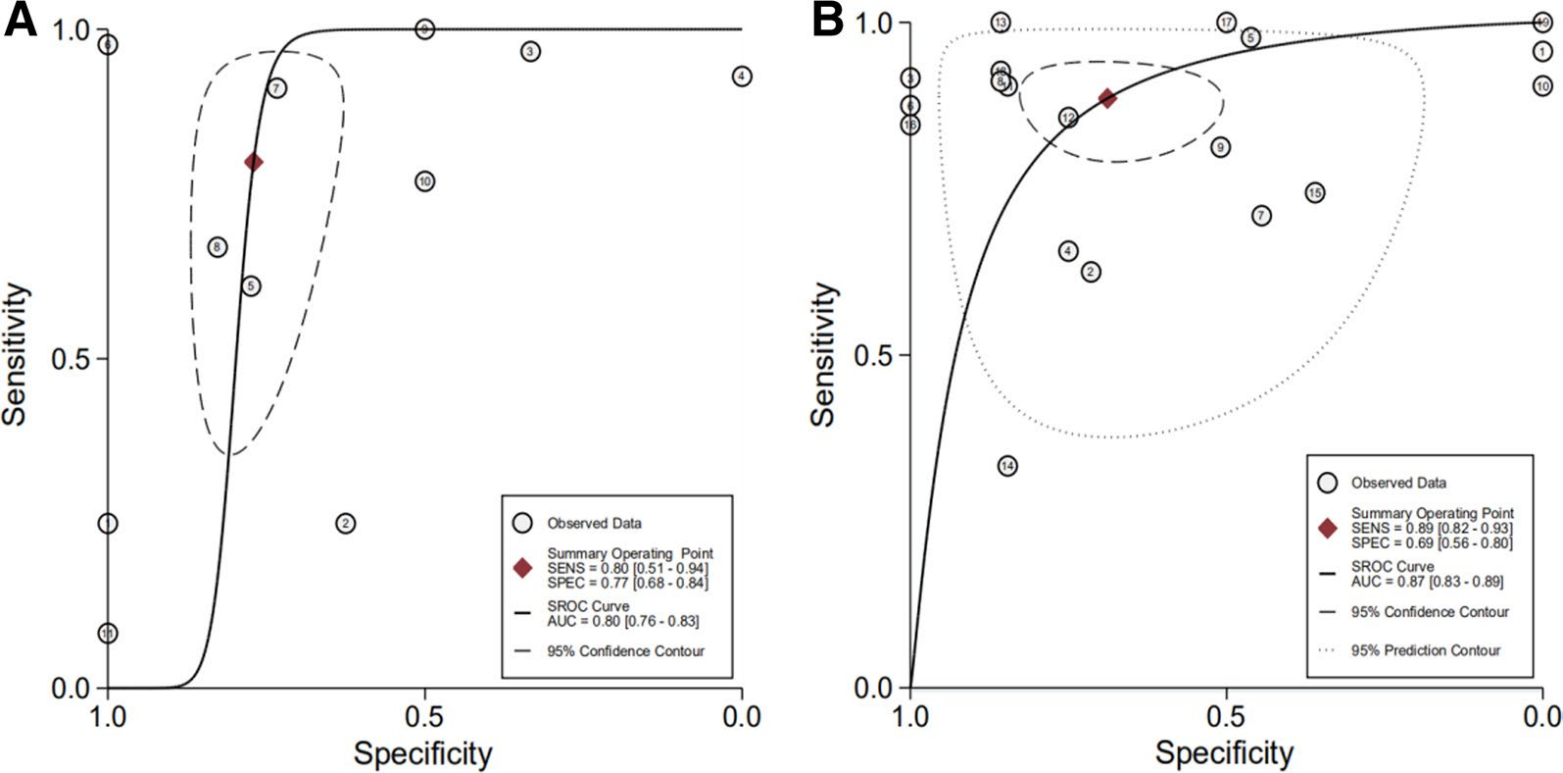
Summary receiver operating characteristic curve (SROC). MRI (**A**), MRA (**B**)

The SROC curve that summarizes the operating points for Se and Sp reveals the diagnostic performance of MRI in detecting ALT, with an area under the curve of 0.80 (95% CI 0.76–0.83; Fig. 4).

**Fig. 5:**
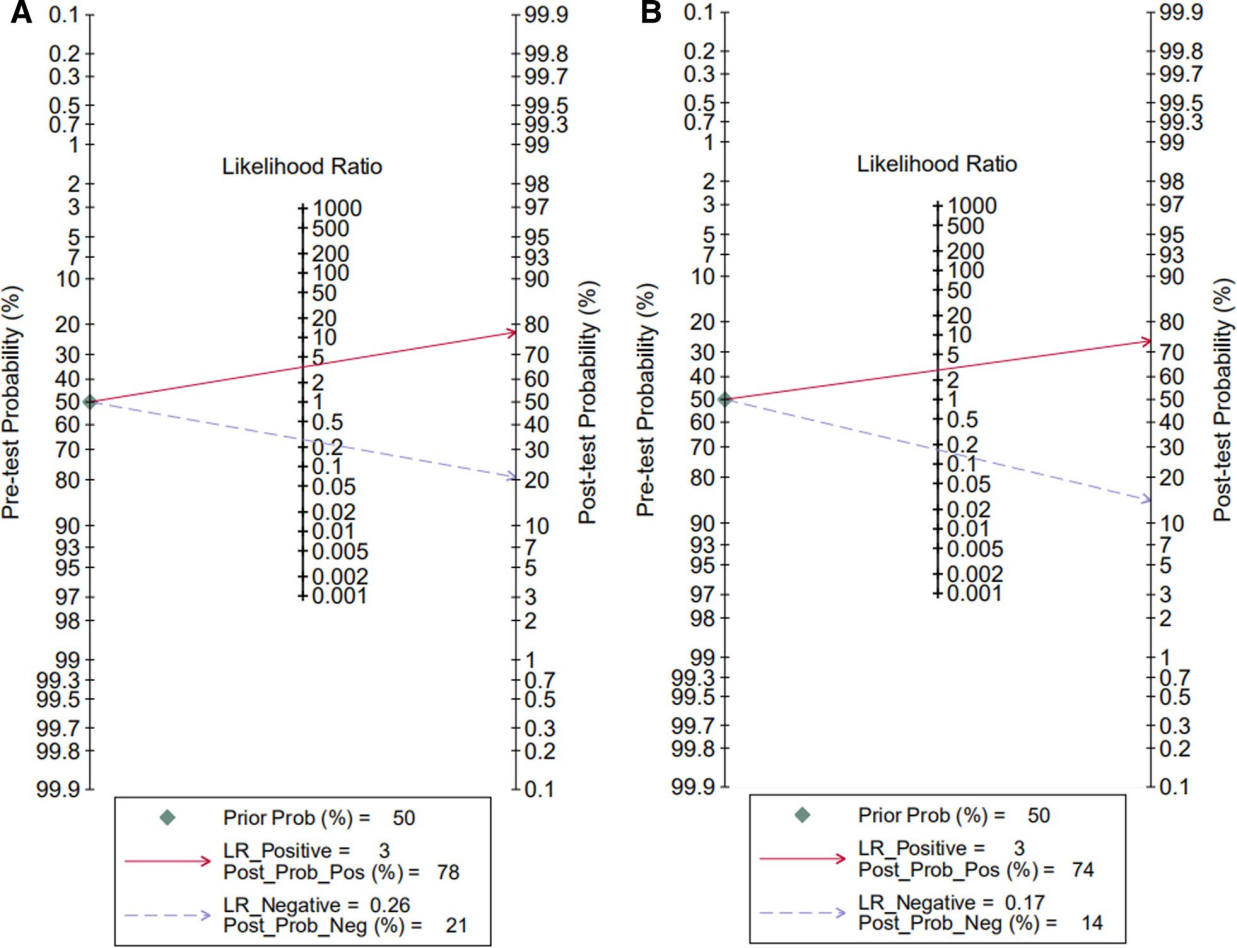
Fagan plots of pre-test and post-test probabilities. MRI (**A**), MRA (**B**)

We calculated the post-test probabilities to determine the implications of MRI for detecting ALT. The plot shows: MRI could increase the post-test probability to 78% in patients and could decrease the post-test probability to 21% in patients with a pre-test probability = 50% (Fig. 5).

### Diagnostic accuracy of MRA for ALT

The summary Se and Sp were 0.89 (95% CI 0.82–0.93) and 0.69 (95% CI 0.56–0.80), respectively. We found significant heterogeneity for both Se and Sp (*I*^2^ = 86.5% and *I*^2^ = 68.0%), which is shown in Fig. 3. The Spearman correlation coefficient was − 0.026 (*P* = 0.915). The heterogeneity might not result from the threshold effects.

The SROC curve that summarizes the operating points for Se and Sp reveals the diagnostic performance of MRA in detecting ALT, with an area under the curve of 0.87 (95% I 0.83–0.89; Fig. 4).

We calculated the post-test probabilities to determine the implications of MRA for detecting ALT. The plot shows: MRA could increase the post-test probability to 74% in patients and could decrease the post-test probability to 14% in patients with a pre-test probability = 50% (Fig. 5).

## Heterogeneity analysis

### Meta-regression analysis

**Fig. 6:**
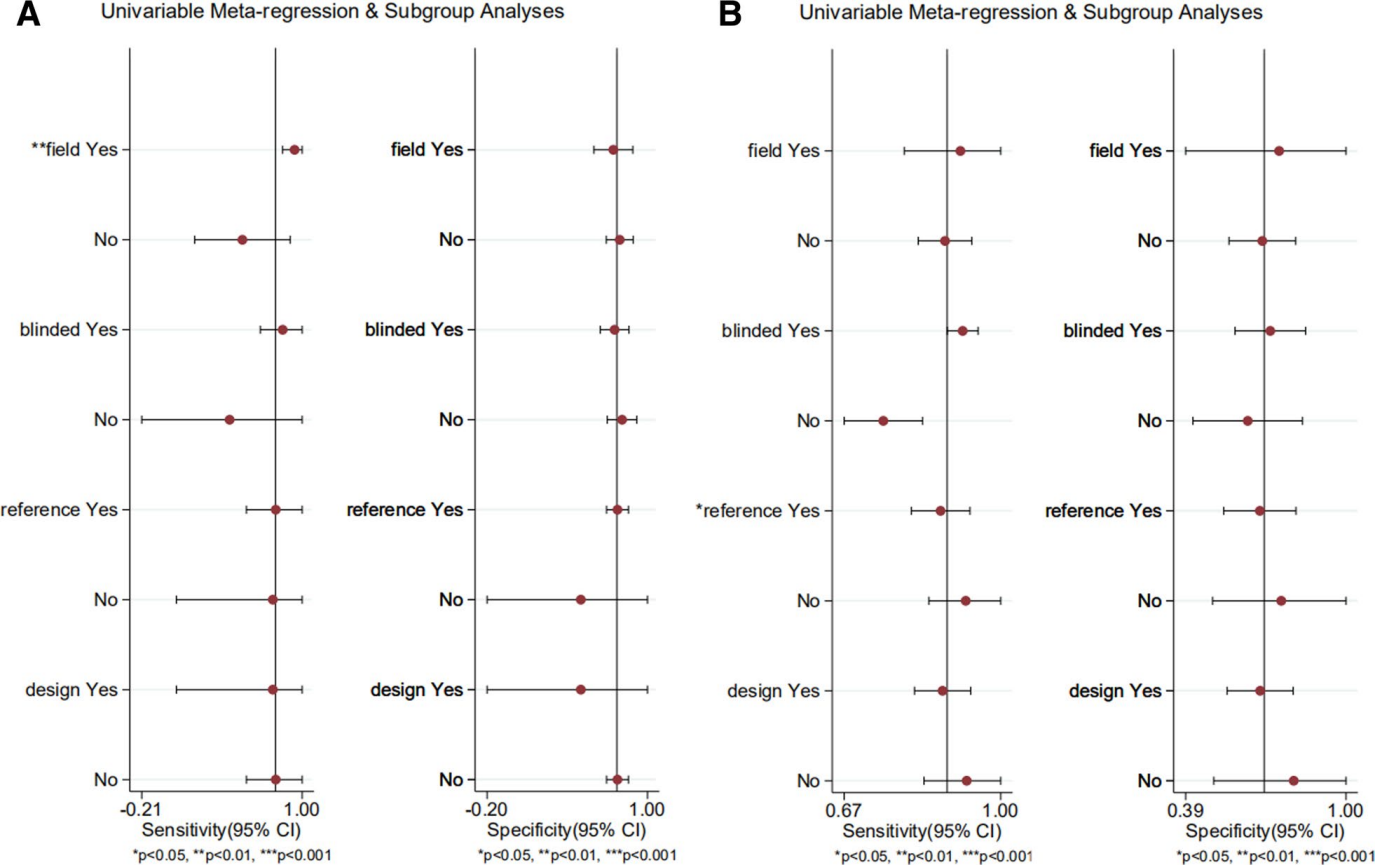
Univariable meta-regression. MRI (**A**), MRA (**B**)

We performed univariate meta-regression to search for the potential sources of heterogeneity (Fig. 6). For Se and Sp, the MR field strength and type of reference standard were significant factors influencing study heterogeneity (*P* < 0.05).

### Subgroup analysis

We performed subgroup analysis to further explore the source of heterogeneity. The results of the subgroup analysis are summarized in Table 3. We cannot conduct a subgroup analysis for the limited number of references of 3.0 T MRA. Regarding the MR field strength, the Se values of 3.0 T MRI were very close to MRA (0.87 vs. 0.89), and the Sp values of 3.0 T MRI were superior to MRA (0.77 vs. 0.69).

**Table 3:** Subgroup analysis

Subgroup	Se (95% CI)	Sp (95% CI)	LR+ (95% CI)	LR– (95% CI)	DOR (95% CI)	Number of studies
1.5 T MRI	0.69 (0.64–0.75)	0.79 (0.67–0.87)	1.64 (0.79–3.40)	0.65 (0.37–1.15)	3.18 (0.84–12.07)	6
1.5 T MRA	0.82 (0.79–0.84)	0.59 (0.52–0.66)	1.90 (1.39–2.58)	0.26 (0.16–0.41)	11.04 (4.75–25.63)	16
3.0 T MRI	0.87 (0.82–0.91)	0.77 (0.64–0.87)	2.40 (1.32–4.35)	0.14 (0.04–0.49)	20.47 (4.56–91.83)	5
3.0 T MRA	NA	NA	NA	NA	NA	2
Reference-1 MRI	0.77 (0.73–0.81)	0.79 (0.70–0.85)	2.07 (1.30–3.30)	0.38 (0.22–0.66)	7.79 (2.87–21.12)	10
Reference-1 MRA	0.81 (0.78–0.84)	0.59 (0.52–0.66)	1.86 (1.33–2.59)	0.30 (0.18–0.48)	8.78 (3.67–21.04)	14
Reference-2 MRI	0.77 (0.62–0.92)	0.50 (0.32–0.68)	NA	NA	NA	1
Reference-2 MRA	0.92 (0.85–0.96)	0.67 (0.45–0.84)	2.64 (1.20–5.84)	0.14 (0.07–0.27)	38.16 (9.84–148.0)	4

Reference-1: Arthroscopy, Reference-2: Arthroscopy and open surgery, NA not available

### Publication bias

**Fig. 7:**
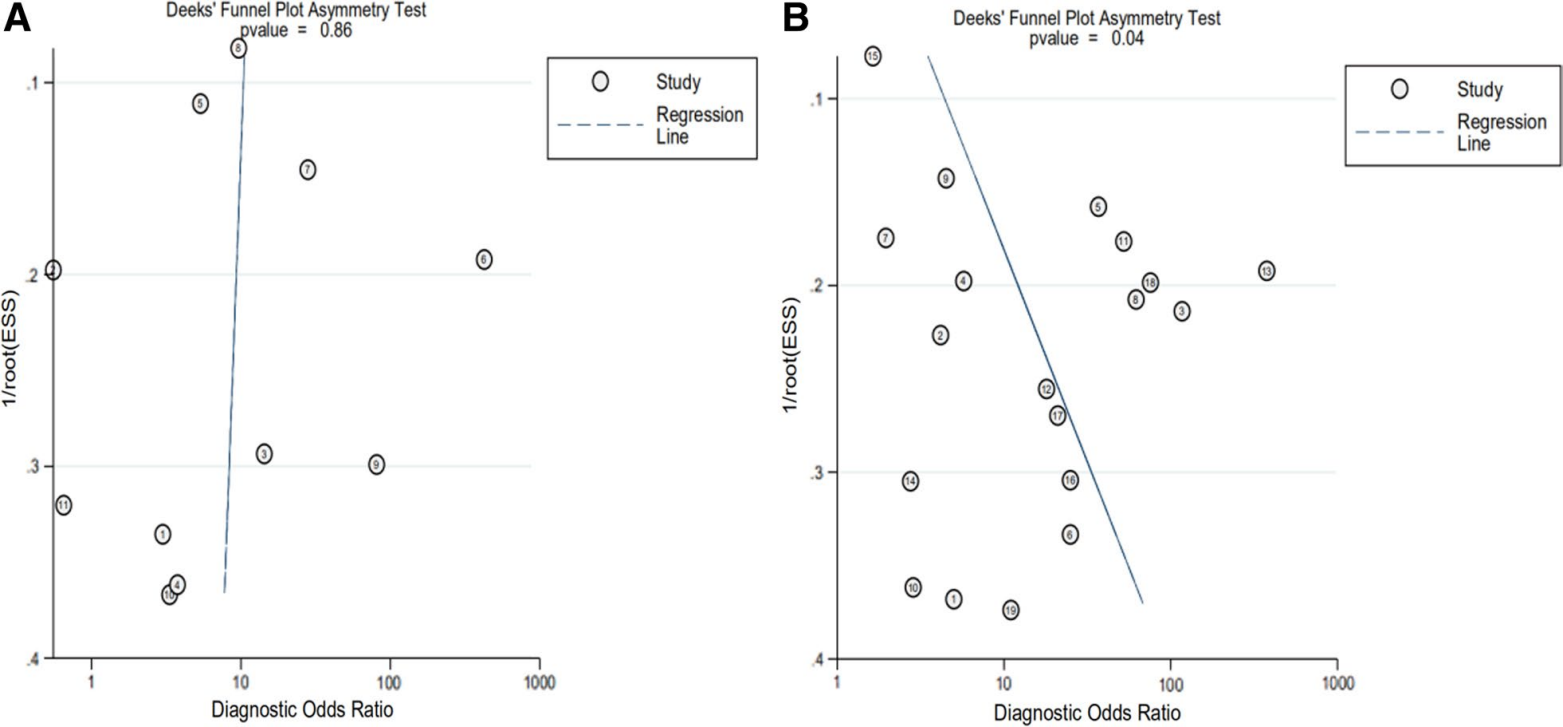
The Deeks’ funnel plot. MRI (**A**), MRA (**B**)

The Deeks’ funnel plot asymmetry test of DOR did not show significant asymmetry (*P* = 0.86, which also showed the absence of a publication bias) in MRI, however, did show significant asymmetry (*P* = 0.04, which also showed the probability of a publication bias) in MRA (Fig. 7).

## Discussion

This meta-analysis demonstrates that MRA has a better performance for detecting ALT than MRI overall, with a pooled Se of 0.89 vs. 0.80, a Sp of 0.69 vs. 0.77, and AUC of 0.87 vs. 0.80. These findings are consistent with the previous three systematic reviews [30–32]. However, the previous systematic reviews did not include sufficient and eligible studies [30–32]. Another interesting finding of our study is that the Se of 3.0 T MRI was very close to MRA, and the Sp of 3.0 T MRI, ability to correctly detect that a patient does not have a labral tear, was greater in 3.0 T MRI compared to MRA. A summary of post-test probabilities also shows: compared with MRA, MRI can help to confirm the suspicious ALT cases. Given that 3.0 T MRI could provide a non-invasive, fast and convenient method to recognize suspicious cases, 3.0 T MRI is more recommended than MRA. So, in clinical practice, clinicians can rely on conventional methods of diagnosis using data from the patients presenting with anterior groin pain, a mechanical hip symptom (clicking, locking, catching, giving way or instability), a positive physical test (such as anterior hip impingement test) and alongside a positive finding on 3.0 T MRI to identify those patients with a symptomatic ALT.

The diagnosis of ALT is a complicated problem for every clinician. No imaging findings, reported symptoms or clinical physical examination findings are ‘stand-alone’ in their ability to diagnosis ALT [33]. Sonography is a relatively inexpensive, quick, non-invasive diagnostic procedure for evaluating ALT, which is however a relatively subjective procedure and relies primarily on the extensive experience of the operator. So far, several studies have assessed sonographic examination for diagnosis of acetabular labral tears, but the validation of this test has been inconsistent [34–41]. Sonographic examination has a lesser diagnostic ability than CTA or MRI/MRA; thus, it is of limited use in clinical practice [34, 35, 41]. CTA is another diagnostic method for evaluating labral tear in patients with claustrophobia, electronic apparatuses, or metallic foreign materials. The diagnostic value of CTA has improved since the advent of multi-detector computed tomography with submillimeter spatial resolution [42, 43]. However, there are only limited data regarding the efficiency of CTA to assess hip labral pathology [14, 35, 42–44], CT also imparts high levels of radiation on the pelvis to young female patients. The patient history and physical findings are important entities to explore in suspicious ALT population alongside diagnostic imaging. A number of physical tests are used to assess ALT, such as flexion-adduction-internal rotation test and flexion-internal rotation test. Up to now, there are 4 systematic reviews aiming to identify the clinical utility of these physical tests, which showed similar results that available physical examination studies were largely heterogeneous, generally of low quality, and did not appear to currently provide the clinician any significant value in altering probability of disease with their use [33, 45–47]. Although the benefits of CTA, MRI, MRA, and US can provide great promise when complemented with physical examination findings, the gold standard of imaging for the diagnosis of ALT has still not been found [30]. MRI is widely used in clinical practice for its excellent soft-tissue contrast advantages. MRI findings are also specific factors affecting surgical decision-making [48]. Therefore, it is necessary and meaningful to clarify whether 1.5 T or 3.0 T is all acceptable for the detection of ALT.

When the clinician is appraising evidence about diagnostic tests they should consider a key concept: how much will different levels of the diagnostic test raise or lower the pre-test probability of disease? So, we calculated the post-test probabilities to understand the clinical utility of MRI/MRA for detecting ALT. Our meta-analysis shows: assuming that the pre-test probability = 50%, MRI could increase the post-test probability to 78% in patients and could decrease the post-test probability to 21% in patients, MRA could increase the post-test probability to 74% in patients and could decrease the post-test probability to 14% in patients. That means MRI may help to confirm the suspicious ALT cases, and MRA may help to rule out the ALT.

Meta-regression analysis revealed that the MR field strength and type of reference standard were significant factors influencing study heterogeneity. Notably, the Se values of 3.0 T MRI were very close to MRA (0.87 vs. 0.89), and the Sp values of 3.0 T MRI were superior to MRA (0.77 vs. 0.69). However, there is insufficient data to summarize the diagnostic value of 3.0 T MRA in subgroup meta-analysis. As we all know, the injection of intra-articular contrast material can play a critical role in the distention of the joint, which may greatly facilitate the radiologist to interpret the MRI. However, MRA is an invasive procedure and carries the risk of joint infection compared to MRI [3]. On the other hand, high field strength magnet can increase the signal-to-noise ratio thus help in a detailed assessment of acetabular labrum [32]. This meta-analysis study, which was the first time to comprehensively evaluate the diagnostic accuracy of 3.0 T MRI, demonstrated a similar ability to detect ALT compared with MRA.

Notably, MRA studies using arthroscopic and open surgery as a reference standard showed higher Se and Sp than those using arthroscopic surgery as a reference standard. The higher diagnostic accuracy in studies using arthroscopic and open surgery as a reference standard might be explained by the blind spots in arthroscopic surgery and additional labral injuries in open surgery. With the reference standard issue being not discussed in the previous three systematic studies [31, 32, 48], further studies still needed to fully assess this issue.

Other possible reasons for the study heterogeneity were: MR sequences (coronal, axial, sagittal, oblique coronal, or oblique sagittal planes), reference test blinded design, the duration interval between MR and surgery, and MR reviewers (single musculoskeletal radiologists, multiple musculoskeletal radiologists or general radiologists). Unfortunately, there was insufficient data to analyze whether the above four potential variables were significant factors influencing study heterogeneity. Furthermore, the combined variability of imaging planes, sequences, slice thicknesses, matrix sizes, resolution, and types of receiver coils were too complex to analyze as subgroup meta-analysis. Park SY et al. compared the diagnostic accuracy of three-dimensional intermediate-weighted fast spin-echo sequence and two-dimensional fast spin-echo sequences for the diagnosis of acetabular labral tears, and they found that Se and Sp were 0.74 and 0.89 for two-dimensional fast spin-echo sequences, and 0.78 and 0.92 for three-dimensional intermediate-weighted fast spin-echo sequence, respectively [49]. 81.8% of included studies mentioned the imaging interpretation was conducted by musculoskeletal (MSK) radiologists. It is generally believed that the accuracy of radiological reporting of hip pathology is based on the training level of the reporting radiologist. McGuire et al. showed that accuracy rates for MSK radiologists were 85% for labral lesions, for community radiologists were 70%, respectively [50]. Of included studies eight presented the results of interobserver reliability, the k-value was all interpreted as above moderate except one study [19]. Freedman BA et al. presented the results of almost perfect intraobserver reliability [5]. Individual assessor variability may have some influence on the diagnostic accuracy of MRI/MRA interpretation. 50% of included studies mentioned the duration interval between MR and surgery, varying from 18 days [13] to > 6 months [5]. This may increase the possibility that the patient labral condition change between the index and reference tests. The reference test blinded design means the findings of the index test were unknown to surgeons. However, the reference test blinded design is impractical in clinical practice. Only one study reported the surgeons were unaware of imaging findings [21].

Kwee RM et al. demonstrated that a sublabral sulcus can be found at any anatomical location in MRI and its prevalence is at least 5% in symptomatic patients [51]. Therefore, MSK radiologists can not be too cautious about sublabral sulcus which usually being misdiagnosed as ALT. Surgeons should also carefully check for acetabular cartilage injury during surgery, as labral tears have been indicated as an adjunctive cause of cartilage injury [52, 53]. Excellent diagnostic criteria are helpful for accurate diagnosis by radiologists. Blankenbaker DG et al. demonstrated that the Lage arthroscopic classification system does not correlate well with the Czerny MRA or an MRA modification of the Lage classification [54]. Constructing a uniform MR imaging criterion to accurately localize a labral tear and define its extent is a vital future research topic. Tiegs-Heiden CA et al. draw an interesting conclusion that gadolinium-based contrast agents may be able to be eliminated from the direct MRA injection without compromising diagnostic accuracy in the hip [55].

### Limitations of this study

Our meta-analysis has potential limitations. First, large heterogeneity was noted between the included studies; although we could perform a meta-regression analysis, we could not fully explain the heterogeneity. Additionally, because of the small number of studies or insufficient data, other potential reasons for study heterogeneity were not included in meta-regression analysis. Second, there did show significant asymmetry in MRA Deeks’ funnel plot, the publication bias may thus influence the reliability of meta-analysis. Third, several subgroup analyses in our investigation were performed on a small number of studies. Additionally, we could not conduct a subgroup analysis for the limited number of references of 3.0 T MRA. Fourth, there was methodological variability in the studies, such as reference standards tests, reference tests blinded design, imaging reviewers, and the duration interval between MR and surgery. The above limitations weaken the generalizability of this meta-analysis's findings to wider clinical practice.

## Conclusion

In Conclusion, MRA has better performance for detecting ALT than MRI overall. Subgroup meta-analysis indicated that the Se of 3.0 T MRI was very close to MRA, and the Sp of 3.0 T MRI, ability to correctly detect that a patient does not have a labral tear, was greater compared to MRA. Given that 3.0 T MRI could provide a non-invasive, fast and convenient method to recognize suspicious cases, 3.0 T MRI is more recommended than MRA. Further randomized controlled studies or prospective studies are still needed to fully assess its diagnostic accuracy.

## Data Availability

Not applicable.

## Abbreviations

ALT: Acetabular labral tears
PRISMA: Preferred Reporting Items for Systematic Review and Meta-Analysis
QUADAS-2: Quality Assessment of Diagnostic Accuracy Studies 2
Se: Sensitivity
Sp: Specificity
MRI: Magnetic resonance imaging
MRA: MR arthrography
CI: Confidence intervals
SROC: Summary receiver operating curve
AUC: Area under the SROC curve
LR–: Negative likelihood ratio
LR+: Positive likelihood ratio
DOR: Diagnostic odds ratio
